# Intelligent Evaluation and Early Warning of Liquidity Risk of Commercial Banks Based on RNN

**DOI:** 10.1155/2022/7325798

**Published:** 2022-05-18

**Authors:** Wei Yan, Yinghua Song

**Affiliations:** ^1^China Emergency Management Research Center, Wuhan University of Technology, Wuhan, Hubei 430070, China; ^2^School of Safety Science and Emergency Management, Wuhan University of Technology, Wuhan, Hubei 430070, China; ^3^Guangxi Vocational College of Quality Engineering, Nanning, Guangxi 530000, China

## Abstract

With the downward pressure of China's economy and the impact of the epidemic, the accumulated market risk has increased the liquidity pressure of the banking industry, and the mismatch between deposit maturity and loan maturity is the main cause for the increase of liquidity risk. The twenty-first century is the era of rapid and in-depth development of data management technology. The explosive growth of massive financial data makes the information data related to the liquidity risk of commercial banks present the characteristics of complexity, diversity, and heterogeneity. The traditional risk early warning model cannot deal with the influence between a large number of influencing factors and the nonlinear factors of commercial bank liquidity risk. Based on this transformation, the circular neural network model is introduced into the field of liquidity risk early warning of commercial banks from the perspective of the mismatch risk of financing maturity of commercial banks, and the driving factors and risk warning signs of liquidity risk of commercial banks are further analyzed from the institutional level, policy level, industry level, and micro commercial bank level. This paper uses network crawler technology, text analysis, and grounded analysis technology to intelligently identify the liquidity risk of commercial banks and establishes an early warning index system based on the influencing factors of commercial banks and internal liquidity risk. Also, it constructs an intelligent early warning model of commercial bank liquidity risk based on deep learning and uses the data of commercial banks from 2000 to 2020 for early warning. The results show that the constructed model has high accuracy, which can provide support for banks and relevant government departments to formulate and resolve bank liquidity risk.

## 1. Introduction

The healthy development of the banking sector plays a decisive role in promoting economic growth and improving people's livelihood. It has outstanding contributions in improving market demand, expanding employment, and increasing taxes. Liquidity risk is one of the main risks of commercial banks. Although liquidity risk, together with credit risk, interest rate risk, exchange rate risk, and market risk, is listed as the main risk for banks to prevent, in fact, the characteristics of concealment, contagion, and hierarchy of liquidity risk are the main causes of financial emergencies. The occurrence of emergencies poses a huge threat to human security and social stability and development and disturbs the normal order of society [1]. With the continuous improvement of people's awareness of crisis, as well as the deepening of the study of liquidity risk and financial crisis, the liquidity risk caused by random factors or systematic errors with negative liquidity risk effect, which can lead to a large area of systemic functional decline or loss of the financial sector, has been continuously valued by scholars from all walks of life. Liquidity risk is a major financial issue for both the central bank and commercial banks. It not only is the deadliest risk for commercial banks, but also has a great impact on the entire financial system and even the real economy. Facing the economic downward pressure and the impact of the epidemic, accumulation of the scale of deposit-loan maturity mismatch increases the possibility of liquidity risk in commercial banks. In order to effectively control the security and stability of financial sectors such as banks, on September 1, 2018, the Beijing Municipal People' s Government prepared the *Beijing Financial Emergency Plan* (hereinafter referred to as the *Beijing Plan*), which is more detailed in terms of liquidity risk attributes and classified disposal. In March 2021, *The Fourteenth Five-Year Plan for National Economic and Social Development of the People' s Republic of China and the Outline for the Vision of 2035* were passed. The outline put forward several requirements for financial reform, and improving the systematic liquidity risk prevention system has become an important task in the new stage. Accordingly, the state has paid more attention to the prevention of systemic liquidity risk.

Through the term conversion function, commercial banks convert short-term funding sources into medium-term and long-term credit funds, which creates a problem of asset-liability term mismatch. The risk of deposit-loan maturity mismatch is the main reason for liquidity risk, which is caused by the mismatch of the use time of investment and financing funds in the time dimension. In the face of the complex external financing environment and regional development differences, how to identify the key factors affecting the liquidity risk of the banking sector and carry out accurate early warning and monitoring before the risk occurs is an important guarantee for the healthy development of the financial market. Therefore, according to the industry characteristics of commercial banks, this paper studies the liquidity risk caused by the deposit-loan maturity mismatch in the banking sector.

Compared with the existing research, this paper makes three changes in the following aspects. Firstly, this paper innovates the banking liquidity risk early warning research perspective. Different from the previous research perspectives that only focus on the internal and external factors of the banking industry, this paper starts from the historical case of the liquidity risk of the commercial bank industry and comprehensively identifies the liquidity risk of the banking industry caused by the mismatch of deposit-loan maturity mismatch under the condition of fully considering the internal and external factors of the liquidity risk of the banking industry. Secondly, this paper innovates research methods of liquidity risk identification in the banking industry. On the basis of the existing risk management theories and methods, the integrated use of web crawler technology, text analysis, and grounded analysis technology can intelligently identify the influencing factors of liquidity risk in the banking industry, expanding the traditional risk factor identification method. Thirdly, this paper constructs an intelligent early warning model of banking liquidity risk. Through deep learning technology, the liquidity risk of banking industry is monitored and the liquidity risk level is determined, which provides the basis for formulating corresponding prevention and control measures.

The structure of the remaining part of the paper is as follows: the second part reviews the relevant literature; the third part defines the relevant samples and data and conducts research design; the fourth part intelligently identifies the liquidity risk of commercial banks and builds an early warning model based on technologies such as crawler and text analysis. The fifth part is the empirical results and analysis; the sixth part is the research conclusion.

## 2. Literature Review

The liquidity risk of commercial banks is caused by deposit-loan maturity mismatch in time dimension, and the research mainly focuses on the following four aspects: first, verify the influence of government intervention and legal level on deposit-loan maturity mismatch risk from the perspective of institutional environment; second, verify the impact of fiscal policy and monetary policy on financing mismatch risk from the perspective of policy environment; third, test the impact of factors such as the level of regional differences in the industry on the risk of corporate deposit-loan maturity mismatch from the perspective of industry environment; fourth, study the impact of mismatch between financing, operation, and investment activities on liquidity risk from the characteristics of commercial banks.

### 2.1. Research on the Impact of Institutional Environment on Liquidity Risk

Institution constitutes the sum of economic, political, and social relations of a country or region [2]. In recent years, more and more scholars have used institutional theory to explain organizational behavior, and the institutional basis view has become the third research perspective on the strategic management of commercial banks after the industrial basis view and the resource basis view [3]. Based on the provincial dynamic panel data, Wang et al. (2016) [4] found that the degree of local political corruption will significantly affect the scale of non-performing loans of banks. But based on the data from the World Bank's 2012 survey on the institutional environment quality of Chinese business operations, Hou et al. [5] found that a certain degree of corruption is conducive to the rational allocation of bank credit resources, but higher degree of corruption will have the opposite effect. As far as government intervention is concerned, Chen et al. [6] pointed out that the level of legal system is conducive to inhibiting government intervention and protecting investment and can guarantee banks and other financial departments to formulate investment and financing strategies in line with enterprise development planning. Zhao (2016) [7] selected data of 30 provinces from 2006 to 2015 and concluded that the higher the level of rule of law, the higher the level of financial development, and the greater the proportion of banks that dare to provide more medium-term loans. Therefore, it can be concluded that the choice of deposit-loan maturity mismatch is an effective tool for poor external legal protection mechanism or high contract execution cost, which is the main reason for the liquidity risk of commercial banks [8].

### 2.2. Research on the Impact of Policy Environment on Liquidity Risk of Financial Sector

Policy impact theory points out that the changes of government economic policy have different effects on the banking industry [9–11]. After the outbreak of the global financial crisis in 2008, domestic and foreign scholars began to explore and reflect on the policy factors behind the outbreak of the financial crisis. The transmission mechanism of government policies on commercial banks is also mainly through fiscal and monetary policies [12, 13]. In terms of monetary policy, a large number of studies have shown that the loose monetary policy with low interest rates implemented by the Federal Reserve for a long time is the trigger for the concentrated outbreak of the financial crisis in 2008. Under the loose monetary policy, in order to expand market share to achieve profit growth, commercial banks gradually relax the approval procedures for housing mortgage loans, and banks continue to improve risk tolerance, thus triggering the financial crisis caused by the accumulation of subprime crisis. Balakrishnan et al. [14] proposed the theory of monetary policy risk-taking channels for the first time. This theory believes that the central bank uses low interest rate monetary policy tools to regulate, reduce the risk identification ability of banks, and improve the risk tolerance of banks. Therefore, banks change their asset pricing and asset allocation and increase their risk-taking level. Brana et al. [15] found that low interest rates encourage banks to invest at high risk and that expansionary monetary policy has a stronger impact on small- and medium-sized banks than large banks. The impact of different types of monetary policy instruments on bank risk-taking is asymmetric [16].

### 2.3. Research on the Impact of Financial Industry Environment on Liquidity Risk

In terms of the environmental impact of the financial industry, Haushalter [17] conducted research based on the diversity in different industries and product markets and drew the conclusion that when the industry is in different cycle stages, it will have different impacts on enterprises to formulate different deposit-loan maturity mismatch strategies; then the financing maturity of enterprises has been in a stable and mismatched cycle of evolution. In response to competition in the banking sector, Gu et al. [18] found that increased competition in the banking sector would weaken the role of economic policy uncertainty in promoting bank insolvency risk and passive risk-taking. The increase in the level of micro competition in the banking sector will significantly weaken the transmission effect of monetary policy bank credit channels (Xue, 2019). In order to improve the level of competition, some banks will deviate from the survival strategy guided by monetary policy, increase interbank lending or issue more credit loans, and increase the probability of liquidity risk. Some scholars have shown that the concentration and competition of the commercial bank market will affect the risk level of industrial banks [19], and more banks adopt radical deposit and loan policy. However, there is still some controversy about how the banking market structure affects the liquidity risk of banks. Repullo [20] also found that lower market concentration was more vulnerable to a fragile banking system in markets facing strict state banking regulation. Agostino et al. provided evidence that the higher bank competition, the better credit conditions for SMEs, by stabilizing bank liquidity risk [21].

### 2.4. Research on the Impact of Commercial Bank Characteristics on Liquidity Risk

Based on the hypothesis of liquidity risk, Diamond [22] incorporated private information into the theory of corporate debt maturity selection, thereby expanding the traditional information transmission model and found that banks with high or low debt levels should maintain shorter debt maturity, while commercial banks with middle debt levels should maintain longer debt maturity. The main reason for the liquidity risk of commercial banks is that enterprises use short-term loans to meet the capital needs of long-term investment. Although some scholars believe that in the developed financing environment in the United States, companies use short-term funds to support long-term investment in this financing term mismatch arrangement can provide liquidity support for corporate investment to ease financing constraints [23]. However, for emerging markets with a high degree of financial containment, this maturity mismatch financing strategy is manifested as a more radical financing behavior, which will increase the liquidity risk of commercial banks. The radical financing term strategy of commercial banks is related to different corporate strategies, corporate creditors, shareholders, and management preferences for debt maturity structure. In order to control credit risk, commercial banks will combine the operating conditions and market expectations of capital investment enterprises to develop long-term and short-term credit delivery strategies [24]. However, due to information asymmetry and agency costs, banks, as credit suppliers, are more inclined to issue short-term loans to strengthen risk control [25]. The characteristics of commercial banks will also affect the liquidity risk. Zhang et al. divided the factors affecting liquidity of commercial banks into external and internal levels, and find total asset, liquidity ratio, capital adequacy ratio, etc., which can significantly affect liquidity risk of commercial banks [26]. Further, based on transaction cost and pecking order financing theory, short-term debt cost is relatively low, and high-quality commercial banks have the ability to bear the flow risk of short-term debt funds [27].

## 3. Research Design

**Figure 1:**
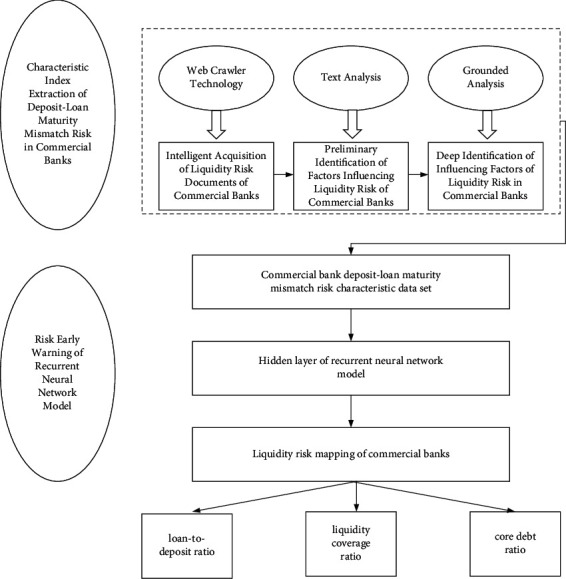
Liquidity risk intelligent early warning process of commercial banks.

In this paper, the risk characteristics of deposit-loan maturity mismatch of commercial banks are intelligently extracted through web crawler, text analysis, and grounded analysis, which are used as the feature data set of the recurrent neural network model for index input. The mapping of liquidity risk level is constructed through the hidden layer to achieve the purpose of risk early warning. Specific research ideas are shown in Figure 1.

### 3.1. Sample Selection and Data Source

The development of capital market plays an important role in the stable operation of China's economy. Referring to the 19 industry categories of listed companies in *The Industry Classification Guidelines of Listed Companies* formulated by the CSRC in 2012, the financial industry listed companies are selected as the initial samples. The accounting information, deposit, and loan data of commercial banks are all from publicly disclosed financial reports and relevant websites such as the People's Bank of China. Therefore, the text collects and sorts out data from CSMAR database, Wind database, Kuroshio website, and securities portal website through crawler technology and manual download. After eliminating the samples with serious missing key variables, 44 listed companies in the financial industry from 2000 to 2020 are finally selected, and a total of 3210 financial industry intelligent early warning samples are collected from the quarterly data of 20 years.

In order to evaluate the generalization ability of the commercial bank liquidity risk intelligent early warning model while adjusting the network parameters, it is necessary to divide the data into training samples, verification samples, and monitoring samples. The intelligent early warning samples of commercial banks' liquidity risk from 2000 to 2019 are selected as training samples and verification samples from the overall sample data. Since the data are reliable enough, this part of the samples is divided into training samples and verification samples of intelligent early warning of commercial banks' liquidity risk by the leave-out method according to the ratio of 7 : 3. Based on the trained commercial bank liquidity risk intelligent early warning model, the commercial bank liquidity risk intelligent early warning samples in 2020 are used as monitoring samples. The distribution of liquidity risk supervisors of commercial banks from 2000 to 2019 is shown in Table 1.

### 3.2. Variable Design

Web crawler technology is used to obtain a wide range of structured and unstructured data about commercial bank liquidity risk early warning, combined with text analysis technology and grounded analysis, to build a unified commercial bank liquidity risk early warning index system.

The purpose of determining the keyword set of news text collection is to search the keywords of the title and text of news information contained in each data source website through the keyword set, so as to screen out the news set involving the liquidity of commercial banks from the mass news database, and prepare for the next research.

Through the analysis of the relevant literature and the characteristics of commercial bank liquidity type news texts, the fuzzy keywords (and keyword derivatives) determined include any word in the set of liquidity-related keywords and any word in the set of risk-related keywords in Table 2.

The data collection uses web crawler technology. Firstly, the debugging tools of browsers such as Chrome are used to summarize the commonness of the target content architecture of each web page. The title and link of the article are obtained by parsing the URL, and the news related to the keyword set is screened. The title and link obtained are classified, and the links are summarized again according to the category. Finally, the text is crawled and written into the file according to the category. Because a large number of CSS selectors and DOM tree structure are needed in the selected scheme, and the Beautiful Soup library in Python is the functional library to parse, traverse, and maintain the *tag tree*. Based on this, Beautiful Soup is selected to complete the crawling and parsing of HTML.

### 3.3. Intelligent Keyword Extraction of Commercial Bank Liquidity Risk Based on Text Analysis

On the basis of using web crawler technology to crawl the text information related to the liquidity risk of commercial banks on each website, it is necessary to analyze the text of this information to achieve the extraction of information on the key characteristics of describing liquidity risk. The dimensionality reduction effect of the feature extraction method is poor, and it is impossible to obtain the effective feature expression of the text, while the text semantic feature extraction method based on the neural network ultimately obtains the word vector, which can better represent the semantic features, and has outstanding performance in the fields of language understanding and sentiment analysis but cannot obtain the actual feature words of the characterization text. Therefore, this paper selects a text semantic feature extraction method based on traditional rules, specifically the TF-IDF method, which considers both the vocabulary frequency in the document and the importance of the vocabulary in the text set. TF-IDF is a kind of word used to assess the importance of a word to one of the files in a file set or a corpus; the algorithm performs keyword extraction of news text and statistically sums the final extracted keywords to obtain a word frequency statistical table based on text keywords, which is calculated as shown in the following formula:

(1) $$\begin{array}{c} TF_{ij}=\frac{n_{ij}}{\sum n_{j}}. \end{array}$$

TF represents the frequency of words that appear in text, where  *n*_*ij*_ is the number of occurrences of the word in text  *d*_*j*_, and the denominator is the sum of the number of occurrences of all words in the text *d*_*j*_.

(2) $$\begin{array}{c} {\text{IDF}}_{i}=\log\frac{\left|D\right|}{\left|\left\{j:t_{i}\in d_{j}\right\}\right|}. \end{array}$$

IDF represents reverse file frequency, where |*D*| is the total number of documents in the corpus. |{*j* : *t*_*i*_ ∈ *d*_*j*_}|  represents the number of files containing word *t*_*i*_. The fewer the documents containing the entry *t*, the greater the IDF, indicating that the entry has a good ability to distinguish categories.

(3) $$\begin{array}{c} TF-\text{IDF}=TF\times \text{IDF}=\frac{n_{ij}}{\sum n_{j}}\times \log\frac{\left|D\right|}{\left|\left\{j:t_{i}\in d_{j}\right\}\right|}. \end{array}$$

Based on the 124572 text data collected by web crawler technology, keyword extraction and word frequency statistics are carried out. After removing the stop words, a total of about 10.32 million words are obtained, but there are many irrelevant words in these words. Then, we use Python's Gensim library to implement TF-IDF algorithm to extract text keywords and filter them, retaining words that are closely related to the liquidity risk of commercial banks. Finally, 3583 keywords were obtained, and high-frequency noise words that were obviously useless for the study were eliminated. Finally, more than 800 risk words were selected. The first 189 words with the highest word frequency were selected as the strongest vocabulary of liquidity risk factors.

### 3.4. Identification of Liquidity Risk Factors of Commercial Banks Based on Grounded Analysis Technology

Based on grounded theory, this paper deconstructs the elements of liquidity risk of commercial banks. Through the study of sample literature, according to the steps of open coding, spindle decoding, and selective coding, this paper analyzes and reviews the induction and expression of liquidity risk of commercial banks in the literature. With the coding function of qualitative research software NVivo12.0, the sample literature is statistically integrated to realize coding, which includes open coding, spindle coding, and selective coding.

**Figure 2:**
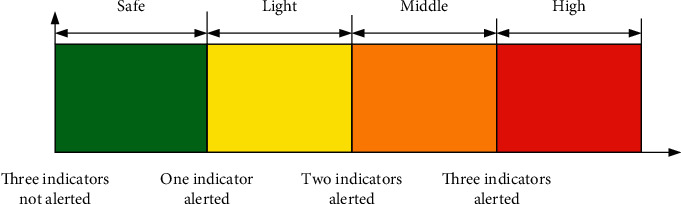
Determination of liquidity risk police level of commercial banks.

Drawing on the classification method of whether listed companies are given warning treatment of delisting risk, and according to the core idea of Basel III strengthening solvency regulation and liquidity regulation to increase the loss and risk resistance of financial institutions, the liquidity risk of financial sector is measured by liquidity coverage rate, deposit-loan ratio, and core debt ratio, and the liquidity coverage rate risk is measured by the ratio of qualified high-quality liquid assets to net cash outflow in the next 30 days greater than or equal to 100%. The ratio of the available stable funds to the required stable funds is less than or equal to 75% to measure the risk of deposit-loan ratio, and the amount at the end of the core debt period and the amount at the end of the total debt period are greater than or equal to 60% to measure the core debt ratio. As shown in Figure 2, the risk level of commercial banks can be divided into four risk warning degrees: safe, light risk, medium risk, and high risk according to different early warning indicators.

The data of relevant macroeconomic indicators such as institutional environment, policy environment, and industry environment are mainly collected through government portals such as the People's Bank of China, marketization index, and the Ministry of Finance. The indicators affecting liquidity risk in commercial banks are mainly collected through the Cathay Pacific or Wind database or combined with the existing research methods.

### 3.5. Linkage Path of Liquidity Risk Factors of Commercial Banks

**Figure 3:**
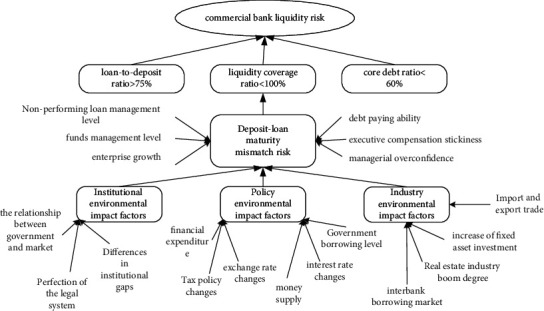
Correlation path of liquidity risk factors of commercial banks.

After passing the theoretical saturation test, this paper outlines the correlation path between the liquidity risk of commercial banks by the internal and external influencing factors of enterprises and then clearly shows the impact path of liquidity risk of commercial banks. As shown in Figure 3, as far as the external environment of commercial banks is concerned, it is the institutional environmental impact factor of government intervention in the market, the difference between the legal system, and the distance between the legal system and the system of commercial banks' business activities; the environmental impact factors of policies such as fiscal expenditure, money supply, and interest rate changes; and the environmental impact factors of fixed asset investment growth and interbank lending. Internally, for commercial banks, it is the factors that affect the mismatch risk of commercial banks' financing terms, such as the management level of non-performing loans, solvency, and stickiness of executive compensation.

Combined with the influencing factors of liquidity risk of commercial banks, as shown in Table 3, the liquidity risk index of commercial banks is constructed as the input layer information of the circulating neural network model of liquidity risk warning of commercial banks.

## 4. Intelligent Early Warning of Liquidity Risk of Commercial Banks Based on Recurrent Neural Network

Commercial banks have many liquidity risk factors and have strong temporal and spatial correlation. The traditional multivariate discriminant analysis, logistic regression model, support vector machine, and other methods are difficult to work in the face of more complex and time-dependent and spatial correlation risk factors, and the fitting effect is poor. Compared with other traditional models, the recurrent neural network model has the function of accommodating high-dimensional feature data sets, processing time series, and extracting time features. Therefore, combined with the characteristics and requirements of risk early warning, this paper uses the circular neural network to construct the intelligent early warning model of commercial bank liquidity risk, the specific model parameters as shown in Table 4, so as to more effectively deal with the temporal and spatial correlation of risk factors, and excavate the time characteristic information, so as to improve the accuracy of commercial bank liquidity risk early warning.

In this paper, there are many commercial bank liquidity risk intelligent early warning training samples, so the stacked cyclic neural network is used to improve the fitting ability of intelligent early warning model of liquidity risk of commercial banks and prevent the problem of under fitting.

Adding the Dropout layer in the recurrent neural network is mainly because it can randomly discard the neurons in the hidden layer with a certain probability, so as to effectively alleviate the overfitting problem and improve the generalization ability of the model.

**Figure 4:**
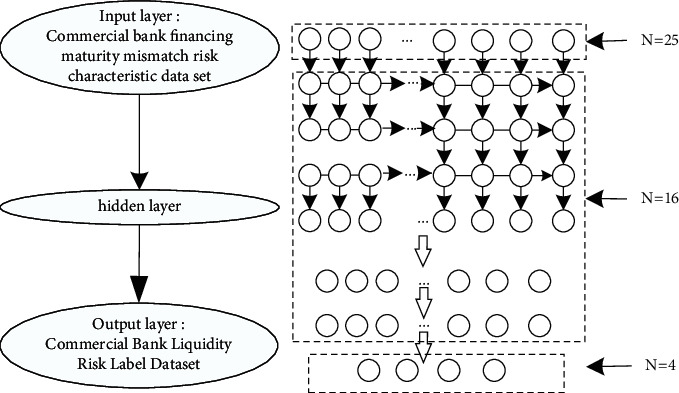
Intelligent early warning recurrent neural network for liquidity risk of commercial banks.

According to the training results, the concrete structure of the recurrent neural network is shown in Figure 4, a five-layer stacked recurrent neural network structure is finally constructed, and a 4-dimensional fully connected layer is added to output the final classification results.

1. Input risk characteristic data: there are 25 liquidity risk indicators in commercial banks, so the input dimension of the first layer recurrent neural network is 25.
2. Hidden layer: in order to better extract the time series information of the liquidity risk of commercial banks and improve the network representation ability, five recurrent layers are used.
3. Hidden activation function: because the intelligent early warning of liquidity risk of commercial banks is a nonlinear system, ReLU function is used to nonlinearly transform the output results at different times to improve the early warning ability.
4. Output layer: the sequence results of the final output of the upper recurrent layer are input to the full connection layer, and Softmax activation function is used to realize the multi-classification division of the liquidity risk alertness of commercial banks. At the same time, cross-entropy loss function is used to update the weight by BPTT algorithm.

Among them, the input risk characteristic data is the commercial bank liquidity risk characteristic data set, namely, the commercial bank liquidity risk index. The hidden layer determines the optimal selection according to the training and validation results. After continuous debugging, this paper finally determines the number of hidden layers with the best optimization and generalization ability. The output layer is the commercial bank liquidity risk label data set, namely, the four alerts of commercial bank liquidity risk: *safe*, *light risk*, *medium risk*, and *high risk*. In order to facilitate the identification and training of the model, one-hot encoding is performed on the risk label data set, where *safe* is represented by (1,0,0,0), while the others are represented as follows: *light risk* (0,1,0,0), *medium risk* (0,0,1,0), and *high risk* (0,0,0,1).

The intelligent early warning model of commercial bank liquidity risk reflects that the recurrent neural network can fully help the transmission of time information, show the dynamic timing behavior, and thus excavate the characteristics of time series information. Through the recurrent traversal of the input data, namely, the time series in the data set of commercial bank liquidity risk characteristics, the characteristics of commercial bank liquidity risk at different times are extracted. At the same time, it is transformed into a higher level and more abstract risk characteristics, and the dimension reduction is realized. Finally, the risk mapping relationship is constructed with the commercial bank liquidity risk label data set. Through the training and learning of the recurrent neural network, the risk characteristics no longer need to be manually extracted, which can automatically expand the factors that play an important role in the liquidity risk index of commercial banks in the risk warning of the deposit-loan maturity mismatch of commercial banks and weaken the irrelevant factors to achieve the organic unity of feature extraction and prediction classification.

**Figure 5:**
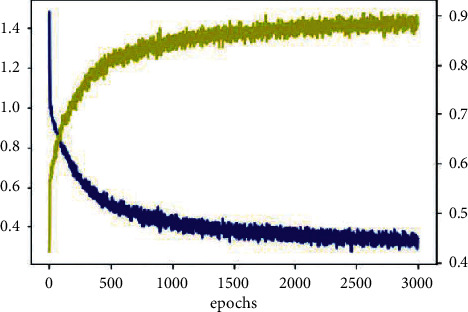
Training effect diagram of intelligent early warning model for liquidity risk of commercial banks.

In the recurrent neural network, through the gradient descent algorithm, specifically, this paper uses the BPTT algorithm to minimize the loss function (CrossEntropy) to achieve weight updating. In order to fully extract the time series characteristics of commercial bank liquidity risk and prevent overfitting problem, this paper sets the number of iterations to 3000.  Figure 5 shows that, after several iterations, the loss of the commercial bank liquidity risk intelligent warning model is finally stabilized at about 0.2, and the accuracy rate reaches 88.21%, which reflects that the model has good fitting degree.

## 5. Evaluation of Intelligent Early Warning Model for Liquidity Risk of Commercial Banks

In order to determine the optimal hyper-parameters of the recurrent neural network, and test the early warning effect of the intelligent early warning model of commercial bank liquidity risk and prevent overfitting problems, it is necessary to test the model with verification samples. Based on a total of 3687 commercial bank liquidity risk intelligent early warning verification samples from 2000 to 2019, the test is carried out.

In order to measure the fitting effect of the intelligent early warning model of commercial bank liquidity risk on the verification set, at the same time, the cross-entropy loss function (CrossEntropy) is used to verify the difference between the output results of this model and the real commercial bank liquidity risk warning. The calculation is shown in the following formula:

(4) $$\begin{array}{c} \text{CrossEntropy}=-\frac{1}{n}\sum_{i=1}^{n}\sum_{k=1}^{k}\left(y_{ik}\right)\log\left(p_{ik}\right). \end{array}$$

The results show that the overall error rate of the commercial bank liquidity risk intelligent early warning model on the verification sample is 0.1397; that is, the prediction accuracy reaches 86.03%. The overall fitting effect of the model is good, and there is no fitting problem.

However, due to the fact that the overall distribution of liquidity risk alerts of commercial banks is left-biased, this means the samples of *medium risk* and *high risk* are less, so in order to further test the prediction performance of the intelligent early warning model of liquidity risk of commercial banks for different alerts, a confusion matrix is constructed, and the precision and recall rates are calculated to realize the statistical results of the sub-alarm, shown in Table 5.

As shown in Table 5, a total of 915 samples of the intelligent early warning model of liquidity risk of commercial banks were tested, and the number of successful predictions of liquidity risk warning of commercial banks totaled 787, of which the number of successful samples of *safe*, *light risk*, *middle risk*, and *heavy risk* was 355, 254, 88, and 90, respectively.

As shown in Table 6, from the perspective of the accuracy rate of the sub-alarm degree, the prediction success rate of the commercial bank' s liquidity risk warning degree of *safe*, *middle risk*, and *high risk* is relatively high, while the prediction success rate of *light risk* is relatively low. Specifically, *safe* and *middle risk* are more likely to be mistakenly classified as *light risk*. From the perspective of the recall rate of the sub-alarm degree, the recall rate of the commercial bank' s liquidity risk warning degree of *safe* is relatively low, while the recall rates of *light risk*, *middle risk*, and *high risk* are relatively high. It shows that the liquidity risk characteristic data set of commercial banks can identify samples with higher risks, which is also in line with the practical needs of commercial bank liquidity risk early warning under the background of high-quality development. For enterprises and regions with risk hidden dangers, risk control and decision-making intervention need to be carried out in time to ensure the sustainable development of the financial industry.

It can be seen that, from the overall test results, the overall accuracy of deep learning in the intelligent early warning of commercial bank liquidity risk is high, and the effect is good. The recognition rate of enterprise samples with high-risk warning is high, which proves the feasibility and scientificity of deep learning technology in the intelligent early warning of commercial bank liquidity risk.

## 6. Conclusion

With the economic downward pressure and the impact of the epidemic, China's financial markets have exposed serious liquidity risk. The liquidity risk of commercial banks is not only caused by internal factors of commercial banks, but also affected by external factors such as institutional environment, policy environment, and industry regional environment. Therefore, how to comprehensively consider the internal and external factors of these commercial banks, based on the use of advanced intelligent early warning model, to realize the intelligent identification of commercial bank liquidity risk, intelligent early warning monitoring, has become an inevitable problem to guide commercial banks to resolve liquidity risk. Using web crawler technology, text analysis method, and grounded analysis as the identification method of commercial bank liquidity risk, and using deep learning technology as an intelligent tool for commercial bank liquidity risk early warning, it is more helpful to explore how to realize the intelligent identification and intelligent early warning monitoring of commercial bank liquidity risk.

Specifically, this paper explores the intelligent identification method of influencing factors of commercial bank liquidity risk and establishes a multidimensional and multi-index early warning index system of commercial bank liquidity risk. This study uses web crawler technology and text analysis to realize the intelligent analysis of commercial bank liquidity risk keywords and uses grounded analysis technology to identify the risk factors that affect the deposit-loan maturity mismatch from the institutional environment, policy environment, industry regional environment, and the characteristics of commercial bank internal financing maturity, so as to establish the foundation for the construction of commercial bank liquidity risk early warning index system; an intelligent early warning model of commercial bank liquidity risk is constructed, which provides a new early warning tool for commercial bank liquidity risk early warning. In this study, the deep learning recurrent neural network model is used to find the mapping relationship between the risk characteristics of commercial bank deposit-loan maturity mismatch and the liquidity risk warning degree of commercial banks, and the early warning model based on recurrent neural network is trained and determined. Through the input of commercial bank liquidity risk characteristic data set, the prediction of commercial bank liquidity risk in the future period is realized, and the timeliness and intelligence of commercial bank liquidity risk early warning practice are improved.

In the early warning of liquidity risk of commercial banks, only the samples of commercial banks in China are selected for research, which makes this study still have limitations. Due to the complexity of the industrial chain, the liquidity risk of commercial banks will also be affected by the financial industry in other countries. In future research, the sample size should be further expanded to comprehensively and reasonably verify the rationality and feasibility of the liquidity risk early warning model of commercial banks constructed in this paper. Secondly, the early warning model for the liquidity risk of commercial banks only uses the recurrent neural network. In the later study, this paper can try to use the recurrent neural network and other evaluation methods of deep learning to compare with each other, compare the differences of various methods to measure the liquidity risk of commercial banks, find out the reasons, and more objectively evaluate the liquidity risk of commercial banks.

## Figures and Tables

**Table 1:** Alarm distribution of intelligent liquidity risk early warning samples of commercial banks.

Risk alertness	Risk intelligence alert sample size	Proportion of samples (%)
Safe	1426	44.45
Light	1112	34.63
Middle	410	12.78
Heavy	262	8.14
Total	3210	100

**Table 2:** Keywords extraction.

Category	Keyword
Keywords related to liquidity risk	Bank, liquidity strategy, deposit channels, loan channels, credit strategy, MBS, subprime mortgage crisis, financial leasing, asset mortgage, credit guarantee, bubble, financing constraints, investment trust
Keywords related to risk	Risk, information asymmetry, irrationality, crisis, term mismatch, structural mismatch, difficulties, constraints, insecurity, contradictions, problems, seriousness, shortage, redundancy, inflation

**Table 3:** Liquidity risk index system of commercial banks.

Goal layer	Element layer	Indicator layer	Variable definition
Deposit-loan maturity mismatch risk of commercial banks	Institutional environmental impact factors	Degree of government intervention	Marketization index report
		Degree of finance marketization	Domestic credits/GDP
			The social financing scale
		Market intermediary organization and perfection of legal system	The perfection of legal system
		The difference between the registered place and the target place of the enterprise	Reflecting differences in policy environment
	Policy environmental impact factors	Financial expenditure	Finance expenditure level
		Taxation variation	Tax policy change level
		Local government debt	Local government borrowing level
		Interest rate changes	Interest rate change level
		Mirror image of foreign exchange reserve	Foreign exchange management level
		Growth rate of m2	Growth level of broad money supply
		Policy uncertainty	Reflecting the level of economic and policy uncertainty
	Industry environmental impact factors	Import and export trade growth	Import and export trade has some relative growth levels
		Interbank offered rate	Lending policy management
		State housing boom index	Reflecting the prosperity of the real estate industry
		Fixed asset investment	Reflecting investment in infrastructure construction in China
	Internal impact factors of banks	Executive compensation stickiness	The stickiness level of executive compensation for enterprise performance
		Managerial overconfidence	Managers' expectations of future market environment
		Enterprise growth	Net assets growth rate
		Amount of non-performing loans in normal loans/normal loans	Non-performing loan management level
		Amount of non-performing loans in concerned loans/concerned loans	
		(General preparation + special preparation + special preparation)/(sub-prime loans + suspicious loans + loss loans)	
		Non-performing loans/total loans	
		Loan amount/deposit amount	Funds management level
		Demand deposit/fixed term deposits	
		Converted funds/deposits	
		Net assets/total weighted risk assets	Debt paying ability
		[(equity + long-term liabilities + operational current liabilities) - long-term assets]/operational current assets	

**Table 4:** Training parameters of liquidity risk intelligent early warning model for commercial banks.

Parameter	Parameter value	Parameter definition
Size	[16，16，16，16，16]	Dimensions of network layers
n	5	Network layers
Activation_ function	ReLU	Hidden layer activation function
Learning rate	0.04	Learning rate, the rate of weight update
Loss function	CrossEntropy	Loss function of cross entropy
Scaling_learning rate	0.001	Learning rate change factor (each epoch), the rate of change for each iteration
Weight penalty L2	0	Weight penalty L2 to limit the weight range
Non-sparsity penalty	0	Non-sparsity penalties are designed to minimize the sum of weights for each layer, i.e., sparsity
Sparsity target	0	Sparsity target, the target value of the sum of weights for each layer
Input zero masked fraction	0	Denoising effect of automatic coding to increase anti-noise ability of network
Dropout fraction	0.4	Dropout network improvement
Epoch	3000	Iteration times
Output	Softmax	Output layer activation function

**Table 5:** Confusion matrix of commercial bank liquidity risk intelligent early warning model.

Confusion matrix		Prediction value of risk alert				Total
		Safe	Light	Middle	High	
Actual value of risk alert	Safe	355	97	6	0	458
	Light	21	254	0	0	275
	Middle	1	1	88	1	91
	High	0	0	1	90	91

Total		377	352	95	91	915

**Table 6:** Evaluation of intelligent early warning model for liquidity risk of commercial banks.

	Safe (%)	Light (%)	Middle (%)	High (%)
Precision ratio	94.33	71.92	92.71	99.45
Recall ratio	77.55	92.32	96.74	98.91
F value	85.12	80.85	94.68	99.18

## Data Availability

The data used to support the findings of this study are available from the corresponding author upon request.
